# Molecular characterization and genetic diversity of *Ornithonyssus sylviarum* in chickens (*Gallus gallus*) from Hainan Island, China

**DOI:** 10.1186/s13071-019-3809-9

**Published:** 2019-11-21

**Authors:** Biswajit Bhowmick, Jianguo Zhao, Øivind Øines, Tianlin Bi, Chenghong Liao, Lei Zhang, Qian Han

**Affiliations:** 1 Key Laboratory of Tropical Biological Resources of Ministry of Education and Laboratory of Tropical Veterinary Medicine and Vector Biology, School of Life and Pharmaceutical Sciences, Hainan University, Haikou, Hainan 570228 China; 2 Norwegian Veterinary Institute, Ullevaalsveien 68 P.boks 750 Sentrum, 0106 Oslo, Norway

**Keywords:** *Ornithonyssus sylviarum*, *cox*1 gene, Identification, Genetic diversity, Chicken

## Abstract

**Background:**

The northern fowl mite (NFM), *Ornithonyssus sylviarum*, is an obligatory hematophagous ectoparasite of birds and one of the most important pests in the poultry industry on several continents. Although NFM poses a serious problem, it remains a neglected pest of poultry in China and other Asian countries. Therefore, a molecular analysis was conducted to provide baseline information on the occurrence, genetic diversity and emergence of NFM in poultry farms from China.

**Methods:**

This study focused on morphological description and identification of adults based on electron microscopy, molecular sequencing of the mitochondrial *cox*1 gene and phylogenetic analysis. We have also used the DNA sequences of the *cox*1 gene to study the genetic diversity, population structure and demographic history. The neutrality tests were used to analyze signatures of historical demographic events.

**Results:**

The mites collected were identified as the northern fowl mite *Ornithonyssus sylviarum* based on external morphological characterization using electron microscopy. Molecular analysis using a 756-bp long partial fragment of the *cox*1 gene revealed 99–100% sequence identity with NFM and phylogenetic inferences showed a bootstrap value of 99% indicating a well-supported monophyletic relationship. Molecular diversity indices showed high levels of haplotype diversity dominated by private haplotypes, but low nucleotide divergence between haplotypes. The Tajima’s *D* test and Fu’s *Fs* test showed negative value, indicating deviations from neutrality and both suggested recent population expansion of mite populations supported by a star-like topology of the isolates in the network analysis. Our genetic data are consistent with a single introduction of NFM infestations and the spread of NFM infestation in Hainan poultry farms and a private haplotype dominance, which suggest that infestations are recycled within the farms and transmission routes are limited between farms.

**Conclusions:**

To our knowledge, this is the first time a molecular report of NFM in chicken from China including other Asian countries using DNA barcoding. The findings have potential implications with respect to understanding the transmission patterns, emergence and populations trends of parasitic infestations of poultry farms that will help for setting the parameters for integrated pest management (IPM) tactics against mite infestations.
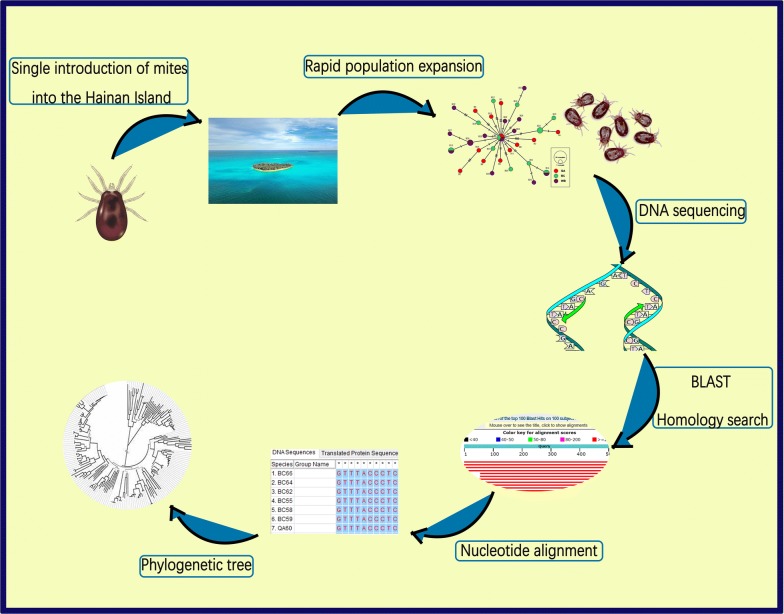

## Background

The northern fowl mite (NFM), *Ornithonyssus sylviarum*, (Canestrini & Fanzago, 1877) (Mesostigmata: Macronyssidae), was first discovered in Pisa, Italy, in 1877 [1], but was first described in substantial detail and under controlled conditions by Sikes & Chamberlain [2]. It is one of the primary blood-feeding ectoparasites in poultry. Many North American records found that 72 species of wild birds from 26 bird families can serve as hosts for NFM [3]. Although NFM and another two species of mites, i.e. the tropical fowl mite (TFM) *Ornithonyssus bursa* and the poultry red mite (PRM) *Dermanyssus gallinae*, are largely considered as avian-specific ectoparasites, when natural host is absent, hungry mites will occasionally feed on a range of mammals, i.e. dogs [4], cats [5], horses [6], and humans particularly in people living or working in close proximity to poultry [7–10]. The poultry red mite *D. gallinae* (Mesostigmata: Dermanyssidae) was first identified by De Geer in 1778 and the tropical fowl mite, *O. bursa* (Berlese, 1888) (Mesostigmata: Macronyssidae) was first recognized as a potentially serious poultry pest by Wood [11]. Tropical fowl mite (TFM) and poultry red mite (PRM) both are also common blood-sucking avian ectoparasites found in the poultry industry [12, 13]. Superficially, NFM resembles PRM in gross appearance; however, there are morphological keys present to differentiate these species [14]. Unlike other mites of poultry, *D. gallinae* feeds mainly at night and for short periods of time. The rest of the time, mites reside in multi-stage colonies in cracks, crevices and hide away from the birds for the remainder of the time [15]. In contrast, NFM and TFM are permanent ectoparasites and complete their entire life-cycle on avian hosts [16, 17]. The nymphs and adults of TFM take blood meals, as opposed to only the protonymph and adult stages in NFM and PRM [2, 18]. NFM can cause severe damage and loss for chicken and turkey breeders, as well as egg degradation, and is considered a key pest for layers in North America, Brazil, China and Australia [19]. Although widespread, NFM is not a major problem in commercial poultry uniformly across the world [19]. PRM has a global distribution and is now at epidemic levels in many parts of the world [15]. The tropical fowl mite is widely distributed throughout subtropical and tropical countries [13]. However, there are some areas like Brazil where all three mite species have been collected from birds [20]. In China, Wang et al. [21] found that NFM prevalence was 47% in parent hens, whilst for the commercial layers the prevalence was 64% for PRM [21]. This suggests that these mites succeed differently depending on the production systems due to differences in their behavior and biology. At high infestation levels, NFM can cause up to 6% blood loss per day per hen, in addition to an activation of the of host’s immune responses. Signs of NFM infestation include restlessness, feather pecking, cannibalism, and anaemia that in severe infestations can cause death [22].

Although exact species identification is an important first step in any biological study, species that are closely related can be difficult to distinguish by the use of morphological characters alone because of similar gross morphology, unclear or inconsistent diagnostic morphological characters and high levels of morphological plasticity [23–25]. More than 40,000 species of mites have been described, and up to 1 million may grace our planet. These eight-legged arthropods (arachnids) represent one of the most successful animal radiations on land [26]. This calls for a need for specialists to be able to differentiate these from thousands of other mite species. Morphological identification may also be very labour-intensive if several specimens need to be identified. Some key characters may also only be present in certain life stages or only in females or males, limiting their usefulness across a variety of sample and sample types. Therefore, the use of sex- and life-stage independent approaches, such as using DNA-based methods could be key for successful identification at the species level. This approach can also provide additional information such as distribution patterns, origin, epidemiological and systematic investigations data for a particular species. Among different DNA regions used in molecular systematics, mitochondrial DNA is a suitable tool as a molecular marker for both intraspecific variation and species identification due to higher evolution rate of genes compared to those in nuclear DNA and their strictly maternal inheritance has been particularly useful at the intraspecific level [27, 28]. As the mitochondrial genome has a high copy number in most cells, molecular diagnostic tools targeting mitochondrial sequences may have the additional advantage of higher detection sensitivity than those targeting nuclear genes [27]. Among different mitochondrial DNA regions, the mitochondrial protein-coding gene cytochrome *c* oxidase subunit 1 (*cox*1) has been used as successful genetic marker in barcoding of mites. Many studies have demonstrated that *cox*1 can provide a powerful tool for phylogenetics at the deepest levels within the Acari [29–31], and for description of more shallow genetic relatedness, such as the movement of mites between farms and to describe industrial routes in larger geographical areas [32]. *cox*1 has been known as the ‘barcoding gene’ and it has proven successful for its use for species identification and discovery across a variety of animal organisms [33]. The usefulness of the marker is justified by the availability of abundant *cox*1 data generated for a variety of organisms in public databases. More importantly, the analysis of intraspecific mtDNA variation can also reveal information about the interconnectivity of populations and past demographic events such as population expansions [34].

China produces more than 40% of all the eggs in the world, making it the worldʼs largest egg producer [35]. These levels of production are associated with the spread of intensive farming systems. The introduction of an ‘enriched cage’ system for rearing chickens could help parasitic mites to better survive and hide as these systems can provide excellent living areas for mite populations [36]. Economic costs have been estimated at €231 million annually due to PRM infestations in European countries alone, with more than 300 million hens in all production types suffering from infestations [37]. It is likely that losses now far exceed that level as an expanding global prevalence of mite infestation is reported [15]. The economic harm caused by NFM is well documented. A study by Mullens et al. [38] in the USA demonstrated that overall reductions in profit were calculated at US$1960–2800 for a 28,000 hen house due to reduced body weight gain, reduced egg weight (up to 2.2%), reduced feed conversion efficiency (by 5.7%) and reduced egg production (up to 4.0%). In addition to these serious welfare issues, mite infestation may significantly threaten the sustainability of food production and undermine the world’s capacity to meet their protein requirements. It is surprising that such an economically important pest for the poultry industry has been largely ignored in Asian countries including China, even from a very basic research standpoint. Similarly, in Europe, PRM research struggles with limited funding, even if there is an urgent need for effective and safe control strategies, sustainable therapy and treatment options, as well as developing solutions for integrated pest management for the poultry red mite control [15]. In order to be able to develop sustainable methods of pest control, we need to be able to understand more about the parasite in question, so that effective solutions, which may control the pests can be implemented. The first stage on this journey is to be able to identify the problem, hence the objectives of this study were specifically aimed towards developing and use of molecular and morphological tools for identification and characterization the NFM ectoparasites recovered from poultry farms in China. This study also sought to describe the intraspecific genetic variability of the *cox*1 gene and assess the population structure of NFM from different localities. A further aim of this study was to use molecular genetic approaches to gain insight into the patterns of emergence and spread of *O. sylviarum* infestation in Hainan poultry farms, providing proof of concept for a novel approach to the study of parasite epidemiology, and eventually supports the formulation of an effective control strategy.

## Methods

### Sampling methods and sampling effort

**Fig. 1:**
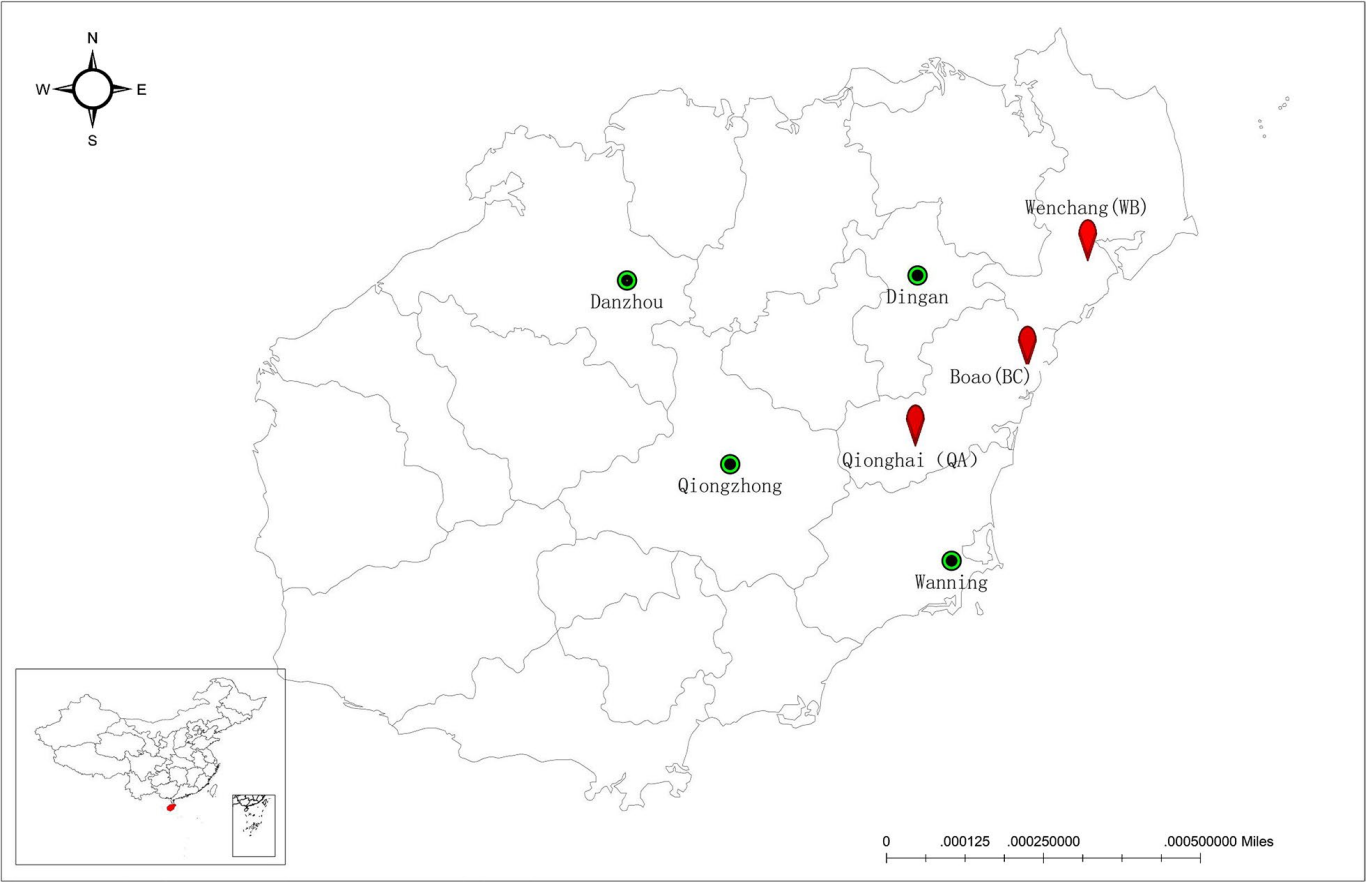
Map of Hainan Island, China showing locations of field sites for mite collections. Red colour indicates positive sites for northern fowl mite *Ornithonyssus sylviarum*

**Fig. 2:**
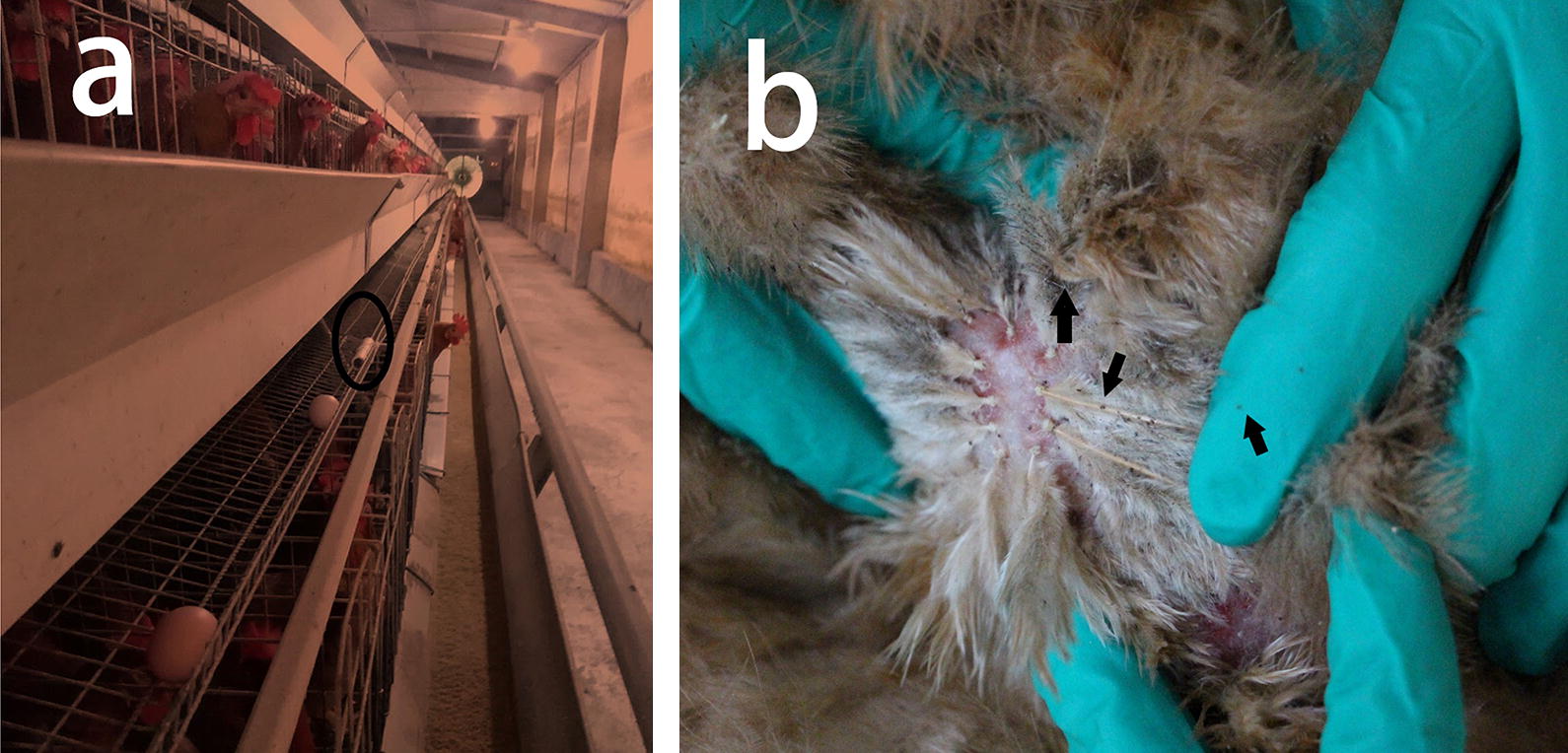
**a** Sampling of mites with trap (within black circle) in an area of mite aggregation, providing fast and easy way for monitoring mite infestations. **b** Northern fowl mites (*Ornithonyssus sylviarum*) on the vent region of an infested roster chicken. Mites are indicated by arrows

The sampling was carried out between May and November 2018, across seven different locations within Hainan Island (Fig. 1): Qionghai; Wenchang; Boao (a town of Qionghai); Danzhou; Wanning; Qiongzhong; and Dingan (for simplicity, the only three positive NFM infested farms reported in the study will be referred to as QA (Qionghai), WB (Wenchang) and BC (Boao). Mites were collected directly from the body surface and vent area of the chickens. Mite traps were used for further sampling of additional mites, with an average of 10 traps placed out in each poultry house. Briefly, the traps consisted of white tubes with inner and outer diameters of 15 mm and 17 mm, respectively, containing rolled corrugated 60 × 70 mm cardboard with thickness of 1 mm [39]. Mite traps were placed as close to chicken level and below feed troughs, inside fittings and fastening clips of cage supports, and under egg conveyor belts for four days (Fig. 2a), after which they were placed into self-sealing plastic bags and sent to the laboratory for mite recovery and then preserved in 70% ethanol at 4 °C until further use. Morphological identification to the species level was carried out using a dissection microscope; when necessary, specimens were slide-mounted, cleared in Hoyer’s medium, identified under a phase-contrast microscope and a scanning electron microscope (SEM) equipped with a digital camera. The identification was based on morphological features [14, 40] in accordance with the morphological keys of Moss [41]. Voucher specimens and corresponding DNA samples were preserved in the HU Vector Biology Lab. For the SEM study, mites were dehydrated in a graded ethanol series (70, 90 and 100%, for 10 min each). Specimens were subjected to critical point drying with CO_2_, sputter-coated with gold using an Anhui Beq SBC-12 coating apparatus, and examined using a field emission scanning electron microscope (S3000N, Hitachi, Berkshire, UK).

### DNA extraction, primer design and PCR amplification

The number of samples for DNA extraction in each farm was set to a minimum 12 mites from each population; QA (*n* = 12); BC (*n* = 12); WB (*n* = 14). Total genomic DNA was extracted from individual mites using a QIAamp® DNA Mini Kit (Qiagen, Hilden, Germany) and protocols therein with some modifications. DNA extraction was carried out following previously described protocols [42, 43], which allow preparation of specimen slides after DNA extraction. Individual mites were placed in a 1.5 ml microcentrifuge tube and the incubation period was extended to 6 hours, for maximum recovery of DNA. DNA was finally eluted in 20 μl of elution buffer and the concentration determined with a NanoPhotometer®N50 spectrophotometer (Wilmington, DE, USA). The exoskeleton of each mite was transferred from the bottom of the tube to the slide with a pipette and mounted in Hoyerʼs medium for morphological analyses. The DNA samples were stored at − 20 °C until used for further analysis. The *cox*1 forward primer OS6F (5*′*-GTG CAG GGA CTG GAT GAA CT-3*′*) was designed in the present study using NFM *cox*1 sequences available on GenBank (FN432541.1; KF218580.1; FN432548.1). Sequences were downloaded and aligned by ClustalW algorithm using the EMBL site [44] and visually checked for conserved regions. Primers targeting the conserved regions of the genes were then designed using Primer-BLAST. Primer properties such as melting temperature, self-complementarity, heterodimer formation, and hairpin structures of the designed primers were further analyzed using Primer-BLAST [45]. The reverse primer CO1R (5*′*-TAC AGC TCC TAT AGA TAA AAC-3*′*) was previously reported [46, 47]. The PCR reactions were performed in a 50 μl volume containing 25 μl of 2xEco Taq PCR® SuperMix (TransGen Biotech, Beijing, China), 1.0 μl of each primer (0.4 μM of each primer), 22 μl water and 1.0 μl of genomic DNA as template. A touchdown cycling protocol was used with the following cycling conditions: an initial denaturation step at 95 °C for 4 min; 10 cycles of 95 °C for 30 s, 62 °C for 30 s (reduced by 1 °C each cycle), and 71 °C for 1 min; followed by 26 cycles of 95 °C for 30 s, 52 °C for 30 s, and 71 °C for 1 min; and a final extension step at 71 °C for 8 min. Amplicons were visualized by UV light in a 1.0% agarose gel following electrophoresis and staining with ethidium bromide. The PCR protocol generated a ≈800-bp fragment. The amplified DNA with expected sizes were excised from gels and purified using a SanPrep Column PCR Product Purification Kit (Sangon, Shanghai, China) according to the manufacturer’s instructions. Purified PCR products were subsequently cloned into the pMD 18-T Vector (TaKaRa, Kusatsu, Japan) and transformed into *Escherichia coli* strain DH5-Alpha competent cells, according to the manufacturer’s instructions. Plasmids from positive colonies were isolated using SanPrep Column Plasmid Mini-Preps Kit (Sangon, Shanghai, China) according to the manufacturer’s instructions and subjected to Sanger sequencing on an ABI PRISM 3730 XL DNA Analyzer, using a BigDye® Terminator v3.1 Cycle Sequencing Kit (Applied Biosystems, Foster City, CA, USA). Both strands were sequenced for all PCR products to ensure good quality of the sequences used in the subsequent analysis. This sequence protocol generated a 756-bp fragment from *Ornithonyssus sylviarum* to be used in the analysis (without primer region).

### Sequencing, phylogenetic and network analysis

The obtained chromatograms were imported into Contig express module in Vector NTI (ThermoFisher Scientific, Waltham MA, USA) and any flanking sequence containing PCR primers were manually removed before sequence alignment and phylogenetic analysis. Forward and reverse chromatograms were assembled in pairs for each sample. First, all sequences generated were aligned by a ClustalW algorithm [48] using the software in MEGA7 [49], and then AlignX module using Vector NTI 9.0 software (Thermo Fisher Scientific). This enabled haplotype assessment, and a total of 32 haplotypes were identified by the guide tree and arbitrarily named (CNFM_1 to CNFM_32). Secondly, additional reference sequences retrieved from GenBank were included in the alignment. All aligned sequences were subjected to phylogenetic analyses using Neighbour-Joining (NJ) algorithms with the Kimura 2-parameter nucleotide substitution model and Maximum Likelihood (ML) algorithms with the Kimura 2-parameter model in the MEGA version 7.0 [49]. For the NJ and ML analyses, robustness of nodes was assessed by including 500 bootstrap replicates. The tree was rooted using a sequence from *Chlaenius pusillus* (GenBank: DQ059793). However, traditional phylogenetic methods have poor statistical power because intraspecific data sets have small genetic distances between individuals [50]. Therefore, relationships between haplotypes were estimated based on the Median-Joining method using PopART [51]. This haplotype network analysis is useful for intraspecific data in revealing multiple connections between haplotypes and indicating possible missing mutational connections. In addition, MEGA version 7.0 was used to calculate numbers of variable sites, singleton sites, parsimony-informative sites and nucleotide composition. Sequences were translated into predicted amino acid sequences using the invertebrate mitochondrial genetic code with publicly available databases using ExPASy tool [52]. Sequences obtained during this study and used in phylogenetic analyses have been deposited in GenBank database under the accession numbers MK993338–MK993375.

### Genetic divergence, diversity, structure, tests of neutrality and estimates of population expansion

To confirm our study in the context of divergence within the species, analyses on divergence of *cox*1 sequence (DNA barcoding marker) were performed using MEGA version 7.0 [49] based on the same model used to infer the phylogenetic relationships. Population diversity indices such as numbers of segregating sites (S), haplotypes number (nh), haplotype diversity (Hd), the mean number of pairwise difference (k) and nucleotide diversity (π) for each location were calculated using DnaSP 4.0 [53]. Haplotype diversity (also known as gene diversity) represents the probability that two randomly sampled alleles are different, while nucleotide diversity is defined as the average number of nucleotide differences per site in pairwise comparisons among DNA sequences [54]. Arlequin 3.5.1 was used to test for population structure of genetic variation within and between populations [55]. The significance of population pairwise comparisons of the fixation indices (*Fst*) was tested by 1000 permutations of the data matrix. The neutrality indices of Tajimaʼs *D* and Fuʼs *Fs* in each population were also calculated using Arlequin 3.5.1 version, with *P*-values generated using 1000 simulations under a model of selective neutrality [55]. Tajima’s *D* uses the frequency of segregating nucleotide sites, while Fu’s *Fs* uses the distribution of alleles or haplotypes [56, 57]. Both tests are based on the principle that a sudden population expansion that is associated with a non-neutral process will show a shift in the allele frequency spectrum compared to a neutral Wright-Fisher model consistent with population expansion under neutral evolution.

### Data analysis

All data were entered in Microsoft Excel 2016. The significance level was set to *P *< 0.05 for all statistical analyses. Molecular diversity indices data were expressed as the mean ± standard deviation (SD).

## Results

### Mite infestations

A total of seven farms were sampled representing a total of 281,000 birds, of which two farms were for parent birds (representing a total of 170,000 birds), five farms were for commercial layer and broiler birds (which covered a total of 111,000 birds). Three farms were infested with NFM representing a total of 120,000 birds, of which one farm was for parent layer birds and the other two were commercial layer farms (Fig. 2b).

### Morphological analysis

**Fig. 3:**
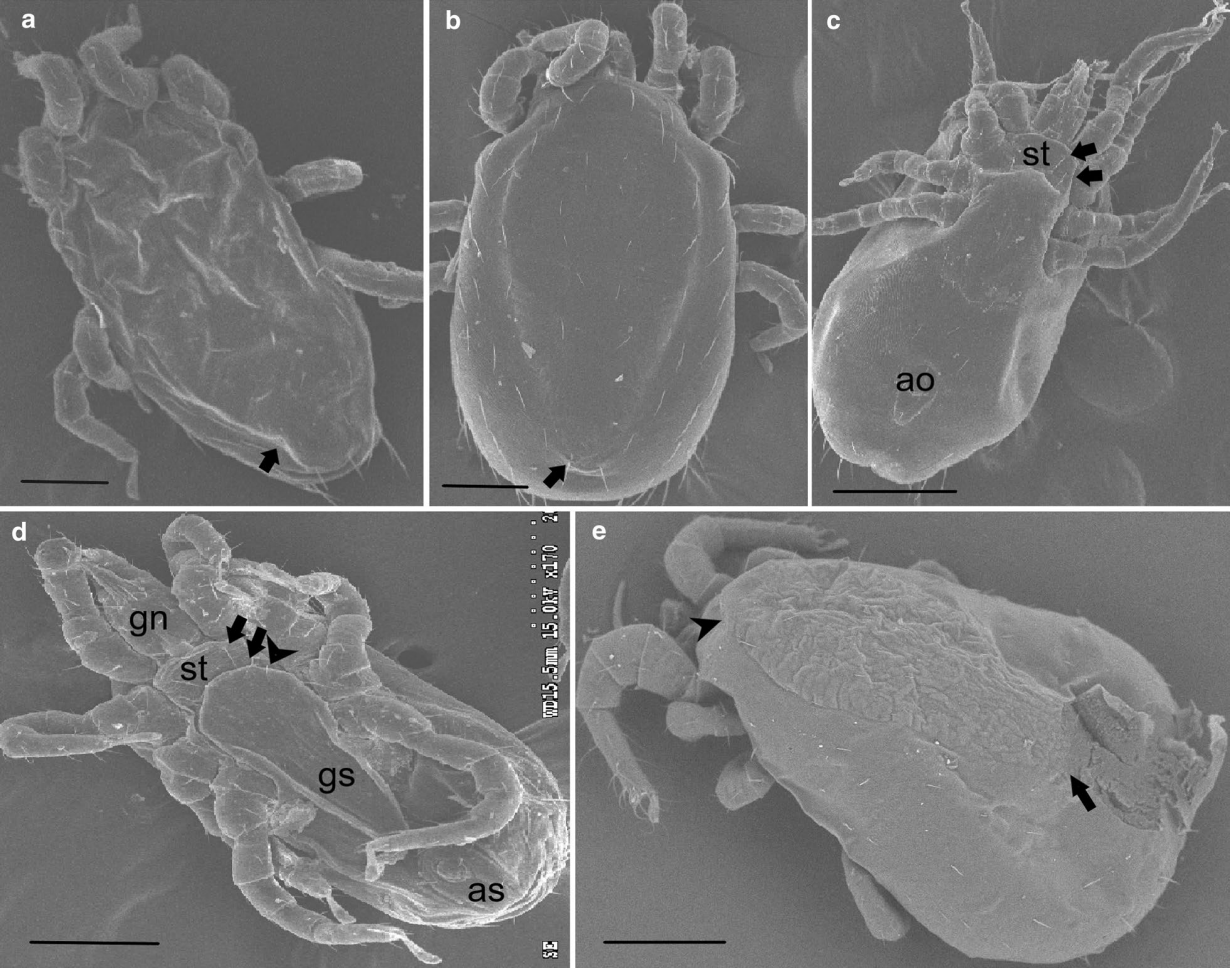
Comparative SEM of dorsal and ventral view of NFM, TFM and PRM. **a** Dorsal view of NFM with a posterior end tapering acutely (arrow). **b** The posterior end tapers more evenly in TFM (arrow). **c** Ventral view of NFM with the genitoventral (epigynal) shield attenuate or narrowly rounded posteriorly and a distinct sternal shield (st) with two pairs of setae (arrows). **d** Ventral view of the NFM with two pairs of setae on the sternal plate (arrows) and a third pair of setae on the unsclerotized integument (arrowhead). **e** Dorsal view of PRM, showing the idiosoma broadly rounded posteriorly (arrow) and prominent shoulder (arrowhead); adults PRM are generally larger in size than NFM and TFM. *Scale-bars*: **a**, **c**, **e**, 300 μm; **b**, **d**, 200 μm. *Abbreviations*: gn, gnathostoma; st, sternal plate; ao, anal opening; as, anal shield

**Fig. 4:**
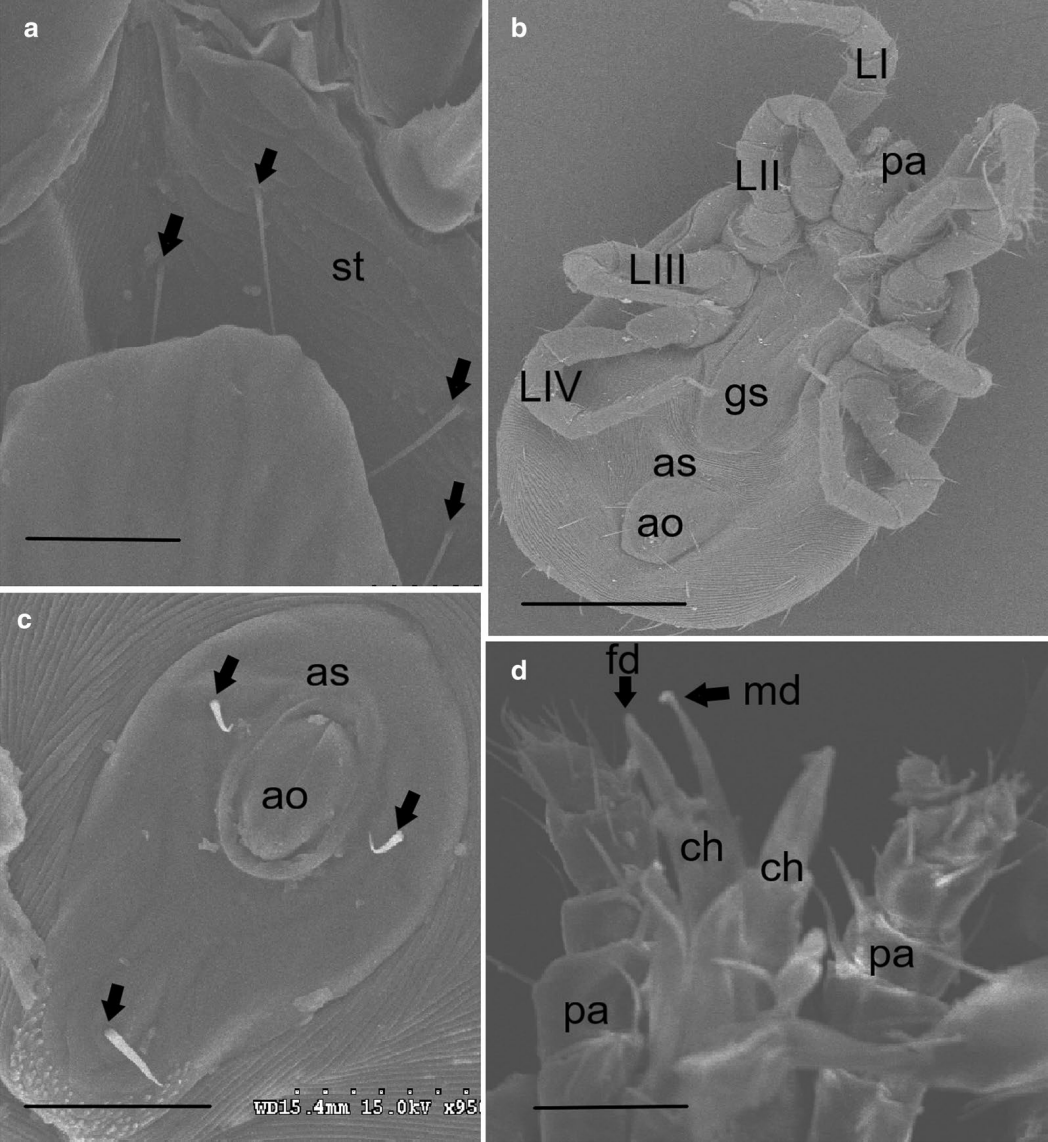
Comparative SEM of NFM, TFM and PRM. **a** Two pairs of setae on the sternal plate of NFM (arrows) whereas TFM have three pairs of setae on the sternal plate (not shown). **b** Female PRM with a keystone-shaped anal plate. **c** The anal plate is distinct, with female NFM being tear-drop shaped with three anal setae (arrows). **d** Female NFM has elongated chelicerae with well-developed and distinct, fixed and movable digit, whereas adult female PRM have a smooth “whip-like” celicerae (not shown). *Scale-bars*: **a**, **c**, **d**, 50 μm; **b**, 500 μm. *Abbreviations*: ch, chelicera; fd, fixed digit; md, movable digit; Pa, pedipalp; ao, anal opening; as, anal shield; st, sternal plate; (LI–IV), leg; gs, genitoventral shield

Although NFM is very similar to TFM in life history and appearance, it can be distinguished by the dorsal plate and number of setae on the sternal shield (Figs. 3, 4). A prominent characteristic for differentiating NFM is that adult females have two pairs of setae on the sternal plate while TFM have three pairs of setae on the sternal plate (Figs. 3d, 4a). The posterior end tapers acutely and the posteriorly narrowly rounded genitoventral shield in NFM (*vs* tapering more evenly in TFM; Fig. 3a–c). Superficially, NFM also resembles another common and economically most important haematophagous ectoparasite of the poultry industry worldwide, the poultry red mite (*Dermanyssus gallinae*). The main feature differentiating NFM from PRM is the shape of the anal plate and the presence of elongate chelicerae in females with movable digit present in NFM but absent in PRM (Fig. 4d). The anal plate of adult females is clearly different, with NFM having a teardrop-shaped anal plate at the terminus of the abdomen and anal opening at the anterior end (*vs* square or keystone-shaped structure of the anal shield in PRM; Fig. 4b–c). Additionally, in PRM the dorsal surface has a shield with prominent lateral margins tapering towards the rear, but these do not reach the distal end of the body and are truncated at the end (Fig. 3e). Measurements (average lengths and widths) of unfed adults of NFM, TFM and PRM were taken to compare sizes. Specimens of *D. gallinae* are generally the largest of the three species. The average lengths and widths of adult unfed mites PRM, NFM and TFM were 900 × 510 μm; 570 × 345 μm and 720 × 465 μm, respectively. Following these data, the mite was morphologically identified as *Ornithonyssus sylviarum.*

### Molecular analysis

DNA concentration on the mite extracts ranged from 13.2 to 38.6 ng/μl. PCR amplification was successful for 99% of the samples. A nucleotide BLAST (megablast/blastn) of the generated sequences in NCBI GenBank database confirmed the identity of the species as all top hits were of *O. sylviarum cox*1 sequences. Four sequences (GenBank: MK993346, MK993351, MK993365, MK993369) showed 100% similarity to the Swedish strain (GenBank: KF218580) whereas the others showed more than 99% identity. The coverage score was however not high, as no available NFM sequences in the database covered the complete regions described in our dataset. There was< 86% sequence identity to *Ornithonyssus bacoti* (GenBank: FM179677.2) and< 80% identity to *D. gallinae* (GenBank: LC029547.1). A combined length of 756 bp of the *cox*1 gene for a total of 38 isolates from three different farms were used to determine intraspecies sequence variability. The overall nucleotide compositions were 42.1% T, 28.7% A, 16.6 % C and 12.5% G. This high A-T content (28.7% A, 42.1% T) is consistent with the general feature of the *cox*1 mitochondrial DNA region in arthropods, and is similar to *cox*1 data from other studies on insects and other mite species [32, 47]. The sequences revealed 55 polymorphic sites, with 59 transitions and 6 tranversions. These polymorphic sites contained 44 (5.82%) singleton variable sites with two variants and 11 (1.45%) parsimony informative sites with two variants at the following positions: 16, 23, 42, 134, 164, 391, 403, 478, 481, 624 and 693. No insertions or deletions were observed in the examined sequences and none of the sequences exhibited any unusual mutations (i.e. false stop-codons, frameshift-causing insertions/deletions). Using the invertebrate translation table, sequences were confirmed to be in frame, and the substitution occurred at the third codon position as expected.

### Phylogenetic and network analyses

**Fig. 5:**
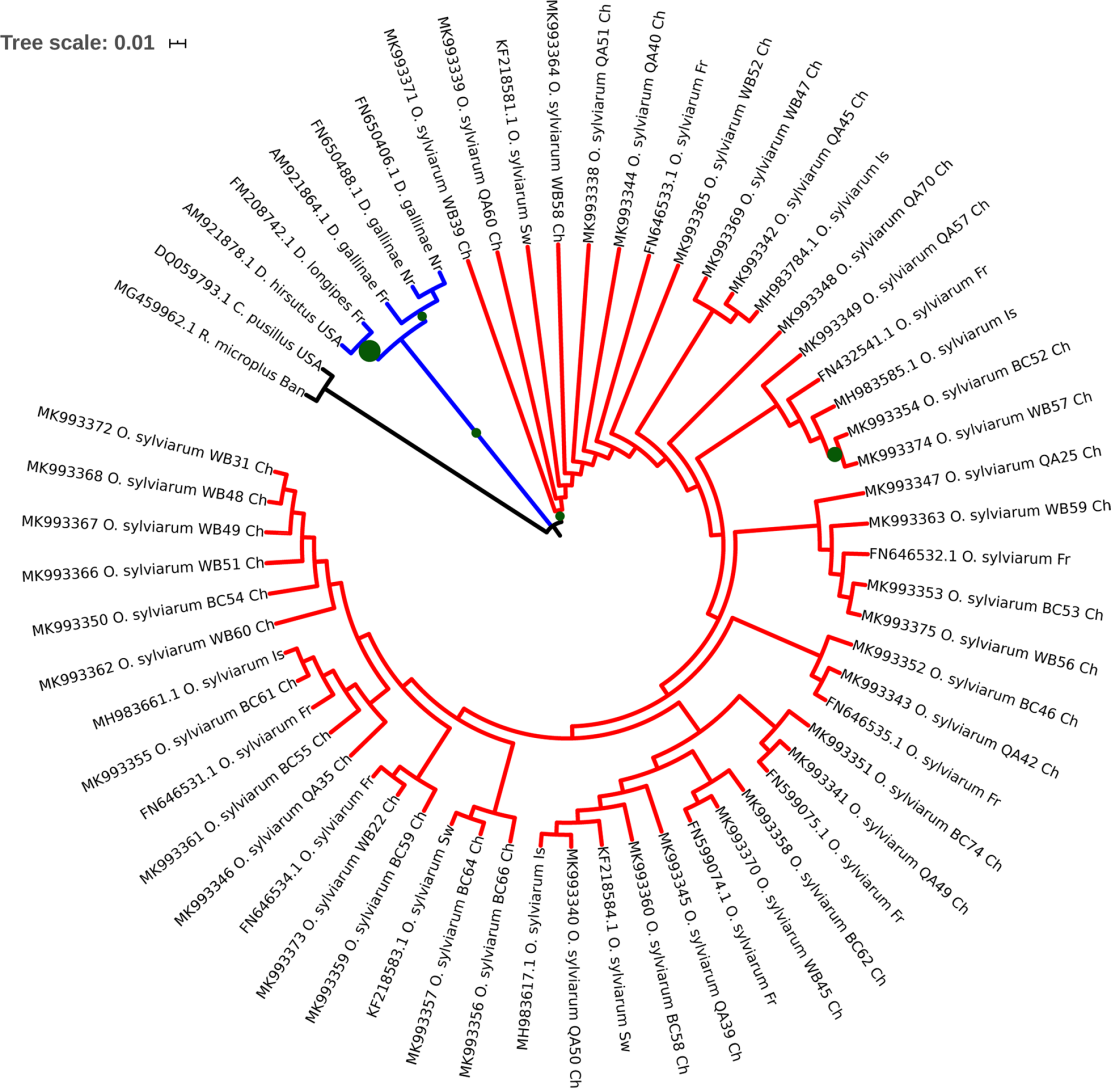
Neighbor-Joining phylogenetic tree based on *Ornithonyssus sylviarum cox*1 gene sequences. Sequence data generated in this study are presented in QA, BC, WB with GenBank accession numbers, species and country names. The optimal tree with the sum of branch length of 0.82084573 is shown. Bootstrap values (> 70%) are displayed as circles on the branches. Circle sizes at nodes are the proportion of 500 bootstrap resamplings that support the topology shown. The tree is drawn to scale, with branch lengths in the same units as those of the evolutionary distances used to infer the phylogenetic tree. The evolutionary distances were computed using the Kimura 2-parameter method and are in the units of the number of base substitutions per site. This analysis involved 60 nucleotide sequences. Codon positions included were 1st + 2nd + 3rd + noncoding. All ambiguous positions were removed for each sequence pair (pairwise deletion option). Evolutionary analyses were conducted in MEGA. Clades are coloured according to the species: *O. sylviarum* (mite: red), *Chlaenius pusillus* (insect: black), *Rhipicephalus microplus* (tick: black) and *Dermanyssus gallinae*, *Dermanyssus longipes*, *Dermanyssus hirsutus* (mites: blue). *Abbreviations*: Ch, China; Ban, Bangladesh; USA, United States of America; Fr, France; Sw, Sweden; Is, Israel; Nr, Norway

**Fig. 6:**
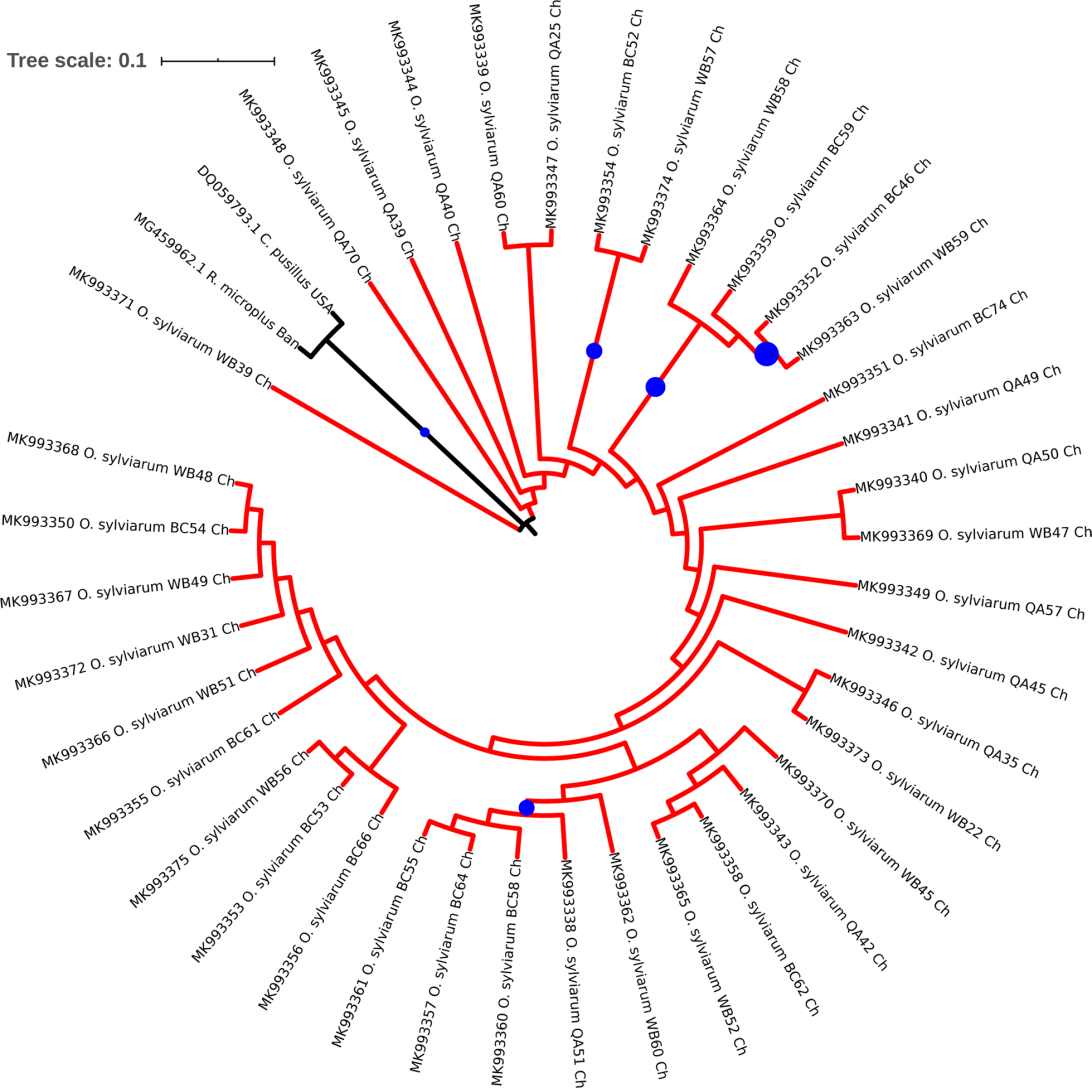
The evolutionary history was inferred using the Neighbor-Joining method based on Chinese isolates generated in this study. Sequence data generated in this study are presented in QA, BC, WB with GenBank accession numbers, species and country names. Bootstrap values (> 70%) are displayed as circles on the branches. Circle sizes at nodes are the proportion of 500 bootstrap resamplings that support the topology shown. The optimal tree with the sum of branch length of 0.43874121 is shown. The tree is drawn to scale, with branch lengths in the same units as those of the evolutionary distances used to infer the phylogenetic tree. The evolutionary distances were computed using the Kimura 2-parameter method and are in the units of the number of base substitutions per site. This analysis involved 40 nucleotide sequences. Codon positions included were 1st + 2nd + 3rd + noncoding. All ambiguous positions were removed for each sequence pair (pairwise deletion option). Evolutionary analyses were conducted in MEGA. Clades are coloured according to the species: *Ornithonyssus sylviarum* (mite: red), *Chlaenius pusillus* (insect: black) and *Rhipicephalus microplus* (tick: black). *Abbreviations*: Ch, China; Ban, Bangladesh; USA, United States of America

**Fig. 7:**
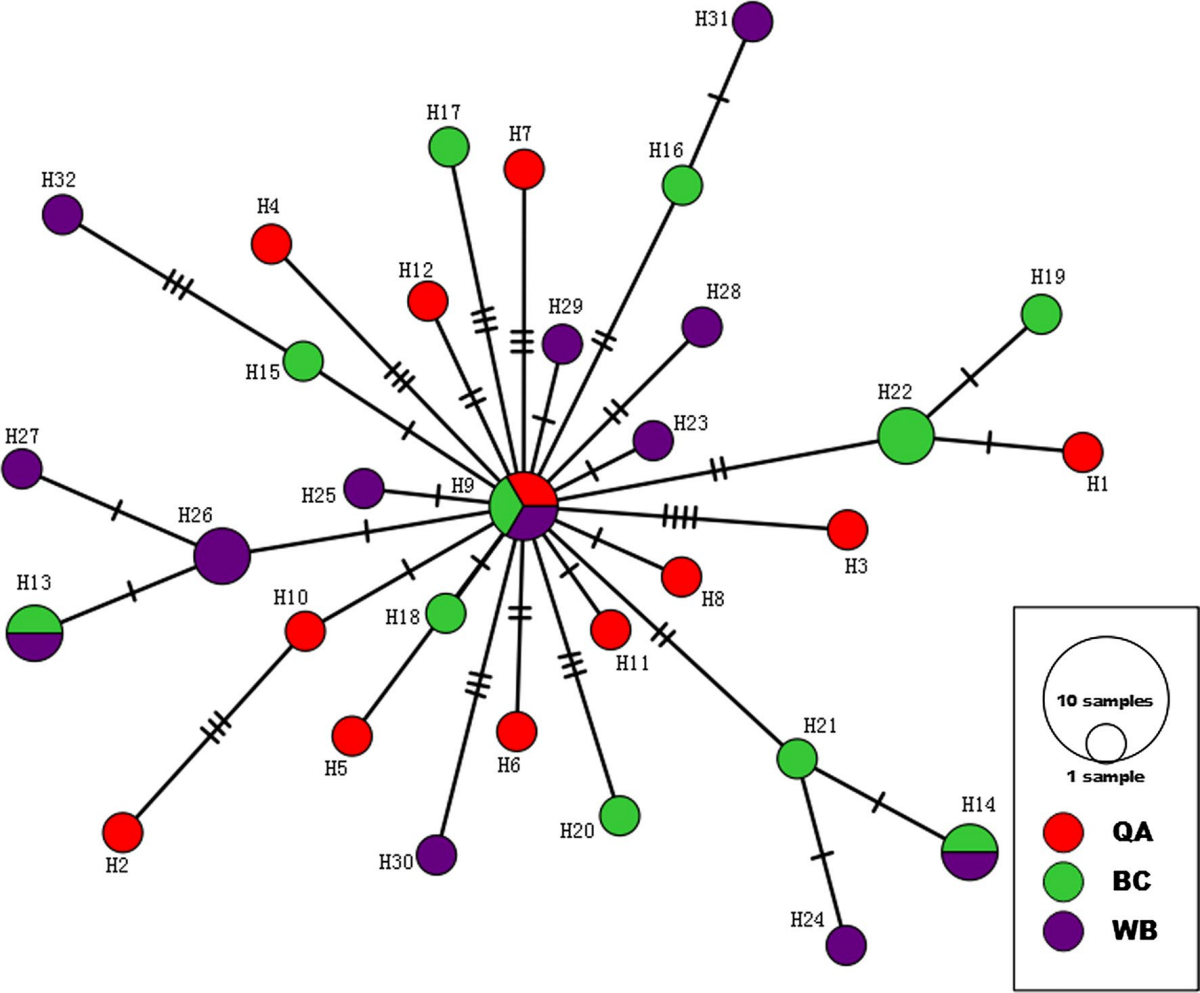
Median-joining haplotype network of mt *cox*1 gene of *Ornithonyssus sylviarum.* Circle size is relative to number of haplotype copies present in the dataset. The most common haplotype (H9) was observed in all populations. A branch represents a single nucleotide change, slashes on branches indicate inferred missing haplotypes (providing a relative estimate of genetic divergence between haplotypes)

An alignment using the shorter GenBank NFM entries included in the phylogenetic calculations, and flanking sites were not included in the analysis. Neighbor-Joining and Maximum Likelihood trees had virtually equivalent topologies. The results of NJ analysis with bootstrap values are shown in Figs. 5 and 6. To further depict the phylogenetic and geographical relationships among the identified sequences, haplotype networks were constructed using the Median-Joining method in PopART software (Fig. 7). The resultant network exhibited a star-like pattern surrounding haplotype H9, which was the most common haplotype in all three populations (QA, BC and WB).

### Population diversity indices and structure

A total of 32 haplotypes were identified in the 38 samples. Among these identified haplotypes, 29 haplotypes were detected as singletons (unique to a single population) and three haplotypes (H9, H13 and H14) were most widely shared. Haplotype 9 was the most common in each population (8.33%, 8.33% and 7.14% in QA, BC and WB, respectively). The relative frequencies of the *cox*1 haplotypes in each Hainan population are shown in Table 1. The number of haplotypes was large in each population. On the other hand, nucleotide diversity was low for each population. The mean haplotype diversity and nucleotide sequence diversity in the three populations were 0.9900 and 0.00521, respectively. Overall, the haplotype and nucleotide diversity were similar between populations. High haplotype diversity (Hd) and low nucleotide diversity (π) are shown in Table 2. The pairwise *Fst* values for genetic differentiation varied from 0.02625 to 0.01299. The pairwise *Fst* values among QA, BC and WB populations were relatively low and with non-significant *P*-values, except for the comparison between QA and WB, indicating very little genetic differentiation. The pairwise *F*st with *P*-values between the locations are shown in Table 3. There was no considerable variation in haplotype diversity (1.00000 to 0.9848) and in nucleotide diversity (0.00569 to 0.004869) in each population, which was reflected in non-significant differences in pairwise comparisons of the *Fst* values.

**Table 1:** Relative haplotype frequencies for three NFM populations

Haplotype number	QA	BC	WB
Hap1	0.0833	0	0
Hap2	0.0833	0	0
Hap3	0.0833	0	0
Hap4	0.0833	0	0
Hap5	0.0833	0	0
Hap6	0.0833	0	0
Hap7	0.0833	0	0
Hap8	0.0833	0	0
Hap9	0.0833	0.0833	0.0714
Hap10	0.0833	0	0
Hap11	0.0833	0	0
Hap12	0.0833	0	0
Hap13	0	0.0833	0.0714
Hap14	0	0.0833	0.0714
Hap15	0	0.0833	0
Hap16	0	0.0833	0
Hap17	0	0.0833	0
Hap18	0	0.0833	0
Hap19	0	0.0833	0
Hap20	0	0.0833	0
Hap21	0	0.0833	0
Hap22	0	0.167	0
Hap23	0	0	0.0714
Hap24	0	0	0.0714
Hap25	0	0	0.0714
Hap26	0	0	0.143
Hap27	0	0	0.0714
Hap28	0	0	0.0714
Hap29	0	0	0.0714
Hap30	0	0	0.0714
Hap31	0	0	0.0714
Hap32	0	0	0.0714
Nt	12	11	13

*Abbreviations*: Hap, haplotype; QA, Qionghai; BC, Boao; WB, Wenchang; Nt, number of haplotypes per population

**Table 2:** Summary of diversity indices based on mt *cox*1 sequences

Population	*n*	S	nh	Hd ± SD	κ	π ± SD
QA	12	25	12	1.0000 ± 0.0340	4.30303	0.005692 ± 0.003411
BC	12	18	11	0.9848 ± 0.0403	3.75758	0.004970 ± 0.003034
WB	14	22	13	0.9890 ± 0.0314	3.68132	0.004869 ± 0.002940
Total	38	65	36	0.9900 ± 0.009	3.93883	0.00521 ± 0.00233

*Abbreviations*: *n*, number of sequences examined; S, number of segregating sites; nh, number of haplotypes; Hd, haplotype diversity; SD, standard deviation; k, average nucleotide difference; π, nucleotide diversity per population; QA, Qionghai; BC, Boao; WB, Wenchang

**Table 3:** Pairwise *Fst* values for *cox*1 sequences of *Ornithonyssus sylviarum* (lower diagonal) and associated *P*-values (upper diagonal)

Population	QA	BC	WB
QA	–	*P* = 0.2060	*P* = 0.0214
BC	0.01299	–	*P* = 0.3818
WB	0.02625	0.00194	–

*Abbreviations*: QA, Qionghai; BC, Boao; WB, Wenchang

### Demographic history

The results of Tajima’s *D* test and Fu’s *Fs* test are presented in Table 4, including the associated simulated *P*-values. Tajima’s *D* values were significantly negative for all populations, indicating an excess of rare nucleotide site variants compared to the expectation under a neutral model of evolution. The results of Fu’s *Fs* test, which is based on the distribution of haplotypes, also showed highly significant negative values for all populations, indicating an excess of rare haplotypes over what would be expected under neutrality. Following this test, the hypothesis of neutral evolution was rejected for all populations.

**Table 4:** Results of Tajima’s *D* and Fu’s *Fs* neutrality tests including associated *P*-values

Population	Tajima’s *D*	*P*-value	Fu’s *Fs*	*P*-value
QA	− 2.13951	*P *< 0.01	-9.03579	*P *< 0.0001
BC	− 1.61094	*P* = 0.0520	-6.93339	*P *< 0.0001
WB	− 1.96944	*P *< 0.05	-9.69639	*P *< 0.0001

*Abbreviations*: QA, Qionghai; BC, Boao; WB, Wenchang

## Discussion

The northern fowl mite, *O. sylviarum* spends its entire life attached to its host, whereas poultry red mites are nocturnal active ectoparasites which typically (though not exclusively) during short visits suck the birds’ blood during periods of darkness and hide themselves in all kinds of gaps and cracks during the daytime [12, 19]. This behaviour calls for different treatment approaches for controlling these mite populations. Correct mite identification is therefore essential for successful integrated control management because dual mite infestation is possible and treatment strategies are likely to differ between these species. In this study, the integration of comparative morphological and molecular methods allowed precise species identification and description of haplotypes from Hainan. Data from the present and previous studies therefore suggest that NFM is geographically dispersed not only in temperate regions but also in tropical areas, and has a wide distribution in China [21]. To the best of the author’s knowledge, this report represents the first molecular evidence of NFM infestation in China. The farm prevalence for NFM infestation in chickens was 42.8%, which is higher than reported in a survey from 11 provinces in China (22.7%) [21]. A range of several factors, including sampling season, diagnostic techniques, poultry management system, biosecurity and housing differences, bird density, poultry rearing system, geography, anthropogenic and environmental factors, may be responsible for the differences in mite population levels observed in different areas of China [58, 59].

It is not surprising that the data in this study reported a high degree of variability within the *cox*1 gene. This molecular marker is known to be highly polymorphic within species, hence its widespread use in analyses of intraspecific variation [60]. According to Avise [34], intraspecific divergences in mitochondrial genes are rarely greater than 2% and most are less than 1%. The genetic distance value obtained from the analysis of the Kimura-2 parameter (K2P) method shows a range of genetic distance values between 0–1.1% (data not shown), suggesting that our result is in the context of divergences within species. This level of intraspecific variation is similar to that found by other researchers for NFM [61]. The pairwise uncorrected genetic distance (p-distance) approach was used by Jansson et al. [61] to estimate sequence variation within the range of 0–2.8%. Neighbor-joining and Maximum Likelihood trees generated in this study indicated a close relationship between the Chinese isolates, as expected when compared with the GenBank entries of NFM from Europe/USA etc. included in the analysis. All the Hainan samples clustered into one group which was supported by a high bootstrap value; this is evidence that the mites studied belong to a single species and that mites from Hainan have a close genetic relationship.

A major finding of this study was the high haplotype diversity and the low nucleotide diversity, indicating only small differences between haplotypes. This was also evident from the Median-Joining network, which showed mostly single nucleotide differences between haplotypes (Fig. 7). In addition, the combination of high haplotype and low nucleotide diversity, as observed in the present study, can be a signature of a rapid population expansion from a small effective population size [34]. The Median-Joining network construction showed a dominant central haplotype (H9) surrounded by several satellite haplotypes; this pattern is considered to represent a recent origin and rapid subsequent population expansion [62]. This finding fits with a major population bottleneck followed by population expansion and the idea that these mites were introduced with a few individuals and then expanded into a much larger population. These results are similar to those described in another feather mite species, *Zachvatkinia isolata* [63], and is consistent with bottleneck or founder event followed by population size expansion during bird-to-bird feather mite transmission. A number of statistical tests have been developed to test selective neutrality of mutations and they are used to detect such population growth [64]. In this study, both tests (Tajima’s *D* and Fu’s *Fs* test) were negative with highly significant *P*-values, particularly for the Fu’s *Fs* test for all populations. The overall negative values resulting from both tests indicate that there is an excess of rare mutations in the populations, which imply a recent population expansion. An analysis including additional neutral nuclear DNA markers could give a more complete perspective on the neutral population structure of these mite populations. Population expansion from a small effective population can be influenced by the following factors. First, the entire life-cycle is short, and large populations can develop rapidly on the birds just after 5 to 12 days [2, 65]. Consequently, weekly doubling of populations is possible under optimal conditions. In addition to movement of the mites by wild birds, the mites can be transmitted by contaminated egg trays, flats, chicken crates and conveyor equipment. Another possible explanation for population expansion might be the result of poor acaricide coverage by spraying and dusting procedures currently used on those farms. These results might explain why NFM maintains a relatively high genetic diversity and a clear signature of historical population expansion. The ability of mite populations to build up quickly means that low mite infestations may rapidly develop into severe infestations debilitating the chickens. An understanding of the demographic history is useful in reflecting population trends in order to integrate multiple control tactics against mite infestations.

In terms of population structure, the *Fst* pairwise values between two populations (BC and WB) were very low (0.00194) with a high *P*-value, three shared haplotypes and four shared branches in the network, indicating no divergence between populations (Table 3, Fig. 7). Similarly, the populations QA and BC showed relatively very little divergence (0.01299) with a non-significant *P*-value, one shared haplotype and one shared branch in the analysis. In contrast, the *Fst* pairwise values between two populations (QA and WB) were relatively high (0.02625) with a significant *P*-value and no shared branches in the analysis, but still shared a common haplotype with the other populations. The distance between the two farms is nearly 120 km (these are the most geographically distant populations in the analysis). For these two populations, the genetic distance value indicated that the geographical distance is one of the factors causing differences in genetic distance values, the farther the distance between populations, the higher the genetic distance value. It is not surprising that the overall *Fst* values are relatively low since they probably came from the same source and a bottleneck population (i.e. all share one common haplotype at the center of the haplotype network and not have any obvious geographically separated clusters in the network) and are still expanding. Furthermore, a star-like gene tree of this part of the network indicates very little geographical structure. Finally, although we identified high genetic diversity, there was little evidence for differentiation between populations, indicating a single panmictic population.

The molecular toolbox is largely unexplored regarding *Ornithonyssus* spp. mites [19]. At present, there have been no reports of *cox*1 gene sequencs for NFM from Asian countries, as all information is originated from European countries, USA or Canada. To our knowledge, the present study reports, for the first time, mitochondrial *cox*1 sequence data for *O. sylviarum* from China which could provide essential information on the genetic structure of NFM populations circulating in Hainan Island. A further aim of the present study was to use novel molecular genetic approaches to gain insight into the emergence and spread of NFM infestation in Hainan. At the farm level, infestation may emerge singly or on multiple occasions through animal movement [66]. The identification of a single common haplotype in all populations suggests a single emergence of NFM infestation on the farms from which the parasitic populations were derived. The statistical Median-Joining haplotype network provided a clear indication that a single introduction of a mite population and a subsequent spread of the parasites has occurred. A similar situation has been reported for the poultry red mite, *D. gallinae*; mostly single haplotypes were found on most farms, suggesting that infestations are recycled within the farms and the transmission routes are few between farms [32]. In contrast, multiple common haplotypes in one population is considered as evidence for multiple introductions of infestation. A recent study reported multiple introductions of *Calicophoron daubneyi* infection in United Kingdom cattle herds, as multiple common haplotypes were identified in 26 populations [66]. It is also noted that there is a chance that our sampling (12–14 mites per farm) may have left some haplotypes undetected, thus underestimating the haplotype diversity per farm. Increased number of samples in some farms in future studies could address this issue. Given that our dataset correctly mirrors the actual diversity per farm, it seems to reflect similar haplotype diversity, and a relatively homogeneous population structure in each farm, which was also found for PRM in Europe [32].

## Conclusions

This study highlights the importance of an integrative approach in taxonomic studies, combining morphology and molecular features. To our knowledge, this study describes for the first time key comparative morphological features for differentiating three mite species sharing the same host species and environment. Our genetic data also provide the first insight into the emergence and the spread of *O. sylviarum* in China. These findings demonstrate a single introduction of a mite population at the farm level followed by extreme population expansion. Our dataset is limited, so further research is needed in order to understand possible transmission routes of this parasite, and to access the significance of its genetic variation and diversity in understanding the epidemiology and biology of this parasite.

## Data Availability

All data generated or analyzed during this study are included within the article.

## Abbreviations

NFM: northern fowl mite
TFM: tropical fowl mite
PRM: poultry red mite
QA: Qionghai
WB: Wenchang
BC: Boao
HU: Hainan University
K2P: Kimura-2 parameter
